# Low-Level Lead Exposure and Intellectual Impairment in Children: Koller et al. Respond

**Published:** 2005-01

**Authors:** Karin Koller, Len Levy, Terry Brown, Anne Spurgeon

**Affiliations:** MRC Institute for Environment & Health, University of Leicester, Leicester, United Kingdom, E-mail: kew13@le.ac.uk; Institute of Occupational Health, University of Birmingham, Birmingham, United Kingdom

We are grateful to Jusko et al. for addressing two concerns raised in our review (Koller et al. 2004) relating to confounding and their use of the Stanford-Binet test in their original report (Canfield et al. 2003). They provide valuable additional analysis of their data, which further support their original findings.

Of relevance to the area of confounding is a recent publication by Mink et al. (2004) in which the effects of different combinations of confounders on multivariate analyses of neurobehavior and neurotoxic exposure were studied. Taking maternal intelligence, home environment, and socioeconomic status as the three most important confounders in this field, Mink et al. urged caution in associating small differences in IQ score in the range of 3–10 points with the effects of environmental exposure; they also made a strong case for a priori consideration and planning for all potential confounders in epidemiological studies. As Jusko et al. explain, this is indeed the method they used in their original analyses (Canfield et al. 2003).

We feel that the weight of evidence across a number of studies has come down in support of an effect of low-level lead exposure on children’s intellectual and neurobehavioral function; as stressed by Bellinger (2004), this evidence is supported by studies on experimental animals in which confounding is largely irrelevant.
